# Does ethnicity have an impact on outcome of CABG?

**DOI:** 10.1186/1749-8090-8-S1-O95

**Published:** 2013-09-11

**Authors:** M El Saegh, A Saleh, H El Saegh, P Yiu

**Affiliations:** 1 Department of Cardiothoracic Surgery, The James Cook University Hospital, Middleborough, UK; 2 Department of Cardiology, Teaching Hospital of University Wurzburg, Germany; 3 Department of Cardiology, The National Heart Institute, Imbab, Giza, Egypt; 4 Department of Cardiothoracic Surgery, New Cross Hospital, Wolverhampton, UK

## Background

Assessment of the impact of gender on long term outcome of CABG.

## Methods

1 year retrospective post-operative analysis of 520 patients who underwent isolated CABG surgery based on morbidity and mortality. The patients were divided into: Caucasians(C); 444 patients and Asians (A); 76 patients.

## Results

Our results showed high significant difference in the Asian group compared to Caucasians for pre-discharge creatinine; p <0.05. There was significant difference concerning post operative arrhythmias “incidence of AF”, p<0.05 in the Caucasians group.

The statistical analysis was insignificant in terms of additive and logistic EuroSCORE, hospital mortality, incidence of post operative infection, post operative renal impairment, ITU readmission, post operative stay, reopening or the need for inotropes.

## Conclusion

Asians are more susceptible to impaired renal functions post operatively; on the other hand Caucasians are more susceptible to post operative arrhythmias so care should be taken to address these complications in each group.

